# Weather Radar Calibration Method Based on UAV-Suspended Metal Sphere

**DOI:** 10.3390/s24144611

**Published:** 2024-07-16

**Authors:** Fei Ye, Xiaopeng Wang, Lu Li, Yubao Chen, Yongheng Lei, Haifeng Yu, Jiazhi Yin, Lixia Shi, Qian Yang, Zehao Huang

**Affiliations:** 1 Changsha Meteorological Radar Calibration Center, Changsha 410207, China; fei.ye@metstar.net (F.Y.);; 2 Meteorological Observation Centre, China Meteorological Administration, Beijing 100101, China; 3 HuaYun METSTAR Radar (Beijing) Company, Ltd., Beijing 100101, China

**Keywords:** weather radar, UAV, metal sphere, radar calibration

## Abstract

Weather radar is an active remote sensing device used to monitor the full lifecycle changes in severe convective weather with high spatial and temporal resolution. Effective radar calibration is a crucial foundation for ensuring the high-quality application of observational data. This paper utilizes a UAV platform equipped with a high-precision RTK system and standard metal spheres to study the principles and methods of metal sphere calibration, constructing a complete calibration process and calibration accuracy evaluation metrics. Additionally, a collocated radar comparison observation experiment was conducted for cross-validation, and metal sphere calibration tests were performed on problematic radars. The experimental results indicate the following: (1) The combined application of a high-precision RTK system and a laser range camera can provide real-time position information on the metal sphere, improving the efficiency of radar target acquisition. (2) The calibration method based on UAV-suspended metal spheres can periodically conduct the quantitative calibration of Z and Z_DR_, achieving calibration accuracies within 0.5 dB and 0.2 dB, respectively, and supports the qualitative inspection of key parameters such as beamwidth and pulse width. (3) During field tests, a high success rate “coarse adjustment + fine adjustment + staring” sphere-finding technique was established, based on automatic switching between RHI, PPI, and FIX scanning modes. This method directs the UAV to adjust the metal sphere to the center of the radar distance bin, reducing the impact of uneven beam filling and bin crossing, ensuring the accuracy of scattering characteristic measurements.

## 1. Introduction

Weather radar is a crucial tool for measuring the occurrence, development, and rapid evolution of convective weather, providing essential support for short-term forecasting. It plays a significant role in meteorological disaster prevention and mitigation, as well as in ensuring the success of major events. As of 2022, China has established a national operational radar network comprising over 240 S-band and C-band weather radars. Networked applications such as multi-radar mosaics and coordinated observations have also developed rapidly. However, due to the complexity of atmospheric conditions and the specificity of radar systems, radar observational data may be affected by various errors and disturbances. Even radars of the same model observing the same target can show inconsistent echoes, severely impacting the accuracy and reliability of quantitative applications. Traditional weather radar calibration methods involve segmental testing and the calibration of various radar subsystems using high-precision instruments. However, “end-to-end” absolute calibration methods based on the entire transmit–receive chain still require further exploration to obtain measurement deviations in actual working environments.

In recent years, researchers have explored the establishment of external calibration systems using metal spheres, antenna far-field calibration towers, ground-based active calibrators, and satellites, combined with conventional instrument testing, forming a rich set of weather radar calibration methods [1,2]. For example, Dutch researchers used metal spheres to calibrate the TARA radar [3], and Swiss-developed radar target simulators (RTSs) have provided high-precision calibration services for key radar metrics in the European weather radar network [4], the US CHILL radar [5], and the Korean PyeongChang Winter Olympics radar network [6], significantly improving the quality of calibrated radars. Given China’s extensive operational radar network, there is an urgent need to establish a portable, economical, and repeatable radar calibration technology. Metal spheres, as standard scatterers, have theoretically calculable radar cross-sections, enabling the absolute true value calibration of the entire radar system chain while minimizing the impact of ground clutter, multipath, and attenuation.

Early work by Williams E.R. et al. used balloons to suspend metal spheres for the end-to-end calibration of radar differential reflectivity, verifying deviations of −0.56 dB and −0.52 dB for 6-inch and 12-inch metal spheres on the KOUN WSR-88D radar [7], respectively. However, this method lacked stability and high precision. Jiapeng Yin et al. used an Unmanned Aerial Vehicle (UAV) carrying metal spheres to calibrate radar antenna pointing and simulate antenna patterns, improving the accuracy of radar constant calculations [3]. Sun et al. described the principles of the external calibration of meteorological radars using UAV-mounted metal spheres, including the selection of standard metal sphere sizes, radar test distances, and calibration steps. They also used Ku/Ka radars to monitor cloud and rain processes and validate reflectivity factors before and after calibration [8]. Zhu et al. achieved differential reflectivity calibration for dual-polarization weather radars using a UAV and metal spheres, showing an average Z_DR_ value of −0.265 dB for 40 cm metal spheres [9]. Li Zhaoming et al. calibrated X-band solid-state weather radars using three different types of standard spheres, showing deviations of 3 dB and 1.7 dB between theoretical and measured values for the reflectivity factor and differential reflectivity factor, respectively [10].

The technological advancements in high-performance multirotor UAVs provide a stable aerial platform for the end-to-end system calibration of weather radars. UAVs carrying metal spheres can fly into the radar’s illuminated area the antenna’s far-field, enabling the calibration of parameters such as the reflectivity factor (Z) and differential reflectivity factor (Z_DR_) [11,12]. However, challenges remain in metal sphere radar calibration, such as large deviations in expected value calculations, the unreasonable selection of sphere material, size, and suspension rope length, non-standard calibration procedures, and difficulty in locating the metal sphere [13,14,15]. This paper utilizes a UAV platform with a high-precision real-time kinematic (RTK) module and standard metal spheres to study the principles and methods of external metal sphere calibration. It designs a complete field calibration process and proposes calibration accuracy evaluation metrics. Through metal sphere external calibration accuracy evaluation and collocated radar comparison analysis, the usability and accuracy of metal sphere calibration are verified, comprehensively demonstrating our in-depth exploration and scientific research achievements in weather radar metal sphere calibration technology.

## 2. Materials and Methods

### 2.1. Theoretical Parameter Calculation for Metal Spheres

#### 2.1.1. Reflectivity Factor

By studying the sphere scattering theory, target signal strength, and radar system detection performance, the weather radar equations based on backscatter cross-section calculations for “point” target equivalent reflectivity factors were derived and analyzed. The specific equations are as follows:

1. Theoretical Value.

σ represents the radar cross-section or backscatter cross-section:

(1) σ=4πβ(π)

Under the Rayleigh scattering condition, the backscatter function is as follows:

(2) $$\beta \left(\pi \right)=\frac{16\pi^{4}r^{6}}{\lambda^{4}}{\left|k\right|}^{2}$$

The radar cross-section σ of a small spherical particle can be expressed as follows:

(3) $$\sigma =\frac{\pi^{5}D^{6}}{\lambda^{4}}{\left|k\right|}^{2}$$

where r is the particle radius (mm); D = 2r is the particle diameter (mm); k represents the absorption coefficient of radar electromagnetic waves by the atmosphere.

The radar cross-section for all particles within a unit volume, known as radar reflectivity (η), can be expressed as follows:

(4) $$\eta =\sum_{i=1}^{N}\sigma_{i}=\int_{0}^{\infty }n(D)\sigma (D)dD$$

n is the number density of particles.

Substituting (3) into (4) gives the following:

(5) $$\eta =\frac{{\pi^{5}\times \left|k\right|}^{2}}{\lambda^{4}}\int_{0}^{\infty }n(D)D^{6}dD$$

Thus, we have the following:

(6) $$\eta =\frac{{\pi^{5}\times \left|k\right|}^{2}}{\lambda^{4}}Z$$

where $Z=\int_{0}^{\infty }n(D)D^{6}dD$.

**Figure 1:**
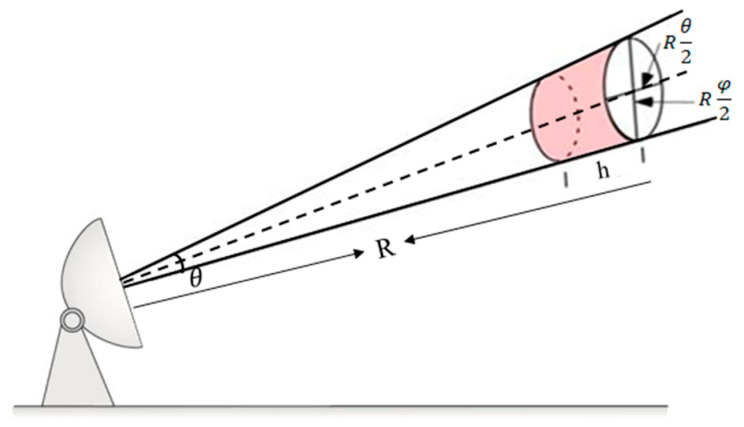
Schematic diagram of effective coverage volume of meteorological radar.

According to the definitions of effective radar illumination depth and effective illuminated volume from the meteorological radar (Figure 1), the calculation for the unit sampling volume can be conducted using the following formula:

(7) $$V=\pi \left(\frac{\theta }{2}R\right)\left(\frac{\varphi }{2}R\right)\left(\frac{c\tau }{2}\right)=\frac{\pi R^{2}\theta \varphi c\tau }{8}$$

In the geometric optics region (λ << D), for a single metal sphere target, it is equivalent to the characteristics of a single precipitation particle during the precipitation process. Therefore, the effective volume reflectivity (η) is equal to the ratio of the backscatter cross-section of a single metal sphere (σ) to the unit resolution volume (V) and is expressed as follows:

(8) $$\eta =\frac{\sigma }{V}$$

Considering that the target is not uniformly illuminated by the radar antenna radiation intensity, a correction value(2ln2) should be added for point-like metal sphere targets [16]. Therefore, by simultaneously combining Equations (6) and (8) and substituting Equation (7), the formula for Z can be obtained as follows:

(9) $$Z=\frac{2ln2\times 8\lambda^{4}\sigma }{\theta \varphi \tau c\pi^{6}{\left|k\right|}^{2}R^{2}}$$

In the equation,

Z—equivalent reflectivity factor ($\frac{mm^{6}}{m^{3}}$)

R—echo distance (m)

λ—radar operating wavelength (cm)

θ—horizontal beamwidth (°)

φ—vertical beamwidth (°)

τ—pulse width (μs)

k—absorption coefficient, different values at different wavelengths and temperatures, the general value is ${\left|k\right|}^{2}=0.93$.

c—speed of light, 2.99792458 × 10^8^ (m/s)

Usually, the equivalent reflectivity factor is represented in decibels (dB), and the calculation formula is as follows:

(10) Z(dBZ)=10×log10(Z)

2. Measured value.

The measured value of the equivalent reflectivity factor observed by the radar for metal spheres can be calculated using the meteorological radar equation. The formula for calculating the peak power of echoes for individual metal sphere targets is as follows:

(11) $$P_{r}^{max}=\frac{P_{t}G^{2}\sigma \lambda^{2}}{({4\pi )}^{3}R^{4}}$$

The formula for the equivalent reflectivity factor observed by the meteorological radar for individual metal sphere targets can be obtained as follows:

(12) $$dBZ=C+P_{r}^{max}+20logR+RL_{at}$$

The formula for the radar constant C is as follows:

(13) $$C=\frac{P_{t}G^{2}\tau c\theta \varphi \pi^{3}{\left|k\right|}^{2}}{1024(ln2)\lambda^{2}}$$

In the equation,

Pr—total power received by the antenna (KW)

Pt—transmit power (KW)

G—antenna gain (dBi)

Lat—atmospheric loss, 0.011 for S-band, 0.019 for C-band, and 0.025 for X-band [17].

#### 2.1.2. Differential Reflectivity

The differential reflectivity (Z_DR_) is the ratio of the horizontal channel reflectivity factor (Z_H_) to the vertical channel reflectivity factor (Z_V_) within the beam volume. For standard metal spheres, the theoretical horizontal and vertical channel reflectivity factors should be equal, resulting in Z_DR_ being 0.

#### 2.1.3. Correlation Coefficient

The correlation coefficient (CC) refers to the degree of correlation between the horizontal and vertical polarization components of the radar-received echoes. It is related to the shape, orientation, and other characteristics of the particles. For standard metal spheres, the theoretical value of the CC is 1.

### 2.2. Far-Field Calibration Methods and Procedures

**Figure 2:**
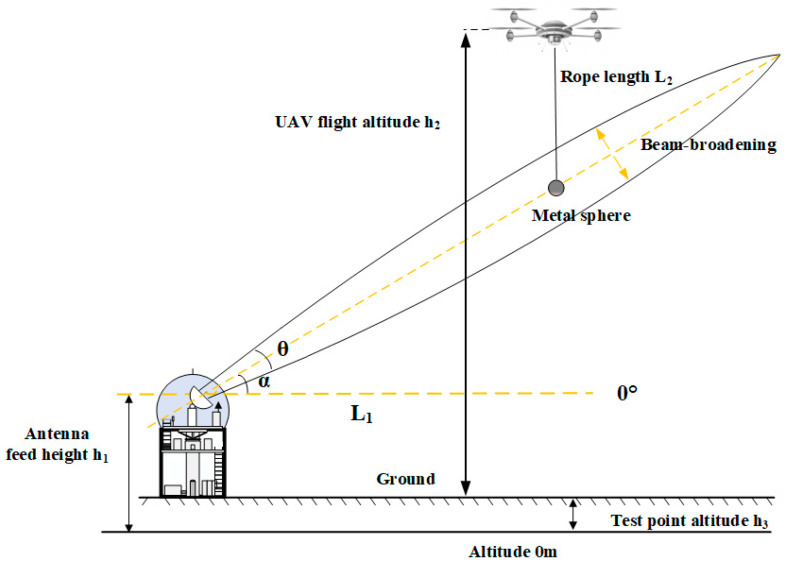
Schematic diagram of calibration using UAV-suspended metal sphere.

Figure 2 shows a schematic diagram of calibration using a metal sphere suspended by a UAV. The radar feed horn altitude is h_1_, the horizontal distance from the radar station to the test point is L_1_, the flight altitude of the UAV relative to the ground at the test point is h_2_, the length of the string suspending the metal sphere is L_2_, the height of the metal sphere is h_2_-L_2_, the altitude of the test point is h_3_, and the distance from the metal sphere to the radar is R.

**Figure 3:**
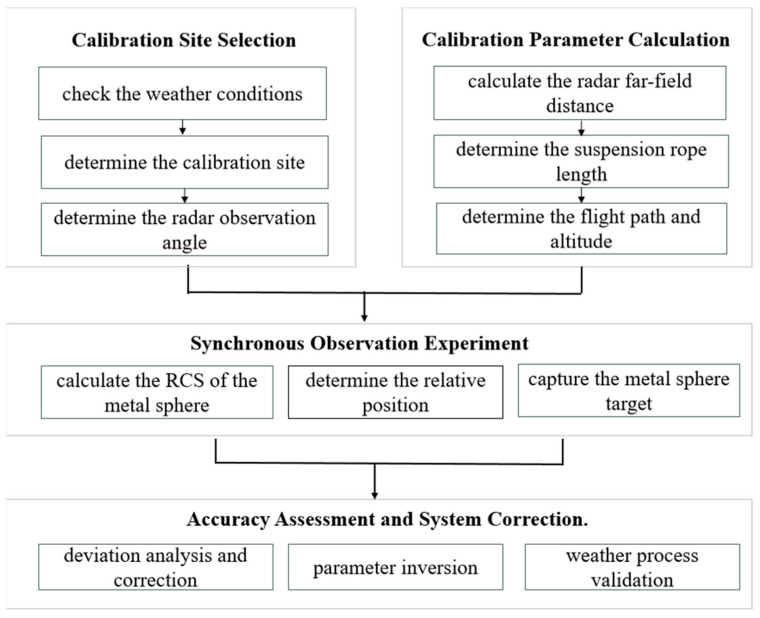
Metal sphere calibration method and procedure.

The weather radar calibration method and procedure based on a UAV-suspended metal sphere mainly include four steps (Figure 3).

1. Site Selection.

Inspect weather conditions and the calibration site, and survey radar ground echoes to determine the testing location.

2. Calculate Theoretical Observation Angles.

Determine theoretical observation angles, select a calibration area outside the radar antenna’s far-field suitable for UAV takeoff, and calculate the relative position between the radar and the metal sphere after determining the rope length.

3. Synchronous Observation Experiment.

Use a high-precision RTK UAV to suspend a metal sphere of appropriate size to the calibration position. Obtain precise coordinates of the metal sphere using a laser rangefinder camera. Simultaneously, after completing routine calibration, set scanning parameters on the radar and sequentially execute Range–Height Indicator Scanning (RHI), Plan Position Indicator Scanning (PPI), and Fix scanning (FIX) modes to efficiently capture and automatically lock onto the metal sphere for effective observation.

4. Accuracy Evaluation and System Correction.

Analyze deviations between observed and theoretical values, assess whether the radar system meets technical specifications, and use deviation results to correct system parameters, achieving comprehensive radar system calibration. Finally, use concurrent precipitation events observed by co-located radars to cross-validate the accuracy of the metal sphere calibration method and procedure.

#### 2.2.1. Site Selection for Calibration

Before conducting field calibration experiments, it is necessary to check the weather conditions, calibration area, and determine the radar observation angle in advance. Select clear weather conditions with no wind or light wind (within 5 m/s), avoiding deviations in metal sphere calibration caused by meteorological echoes and the external environment, far from electromagnetic interference, high-voltage lines, and other hazardous areas. Select the best calibration location that meets the conditions for UAV takeoff, and apply for airspace protection for that area. Check the radar echo map under clear sky conditions, select the direction without environmental noise and ground clutter interference outside the far-field range, and use it as the best angle for the radar observation of the metal sphere.

#### 2.2.2. Calibration Parameter Calculation

1. Calculate Far-Field Distance.

When observing in the far-field of the antenna, it is approximately considered that the target is illuminated by plane waves. The influences of electromagnetic wave reflection and diffraction on the measurement are relatively small, and greater distances can improve the signal-to-noise ratio between the target and the background. Therefore, to ensure measurement accuracy and reliability, metal sphere calibration is typically conducted in the far-field region of the antenna. The formula for calculating the minimum far-field distance is as follows [18]:

(14) $$L=\frac{2D^{2}}{\lambda }$$

where L is the minimum far-field distance, D is the diameter of the radar antenna, and λ is the wavelength of the radar. Table 1 provides the minimum far-field distance values for different antenna diameters corresponding to typical frequencies in different frequency bands.

2. Determining the Length of the Suspension Rope.

According to the latitude and longitude of the radar station and the metal sphere, the straight-line distance R between the metal sphere and the radar station can be calculated. The formula is as follows:

(15) $$R=R^{\prime }\mathrm{arcos}(\sin(\mathrm{lat}1)\times \sin(\mathrm{lat}2)+\cos\;(lat1)\times \cos\;(lat2)\times \cos\;(lon1-lon2))$$

where A(lat1,lon1) represents the latitude and longitude of the radar station, B(lat2,lon2) represents the latitude and longitude of the metal sphere, and R′ represents the radius of the Earth.

**Figure 4:**
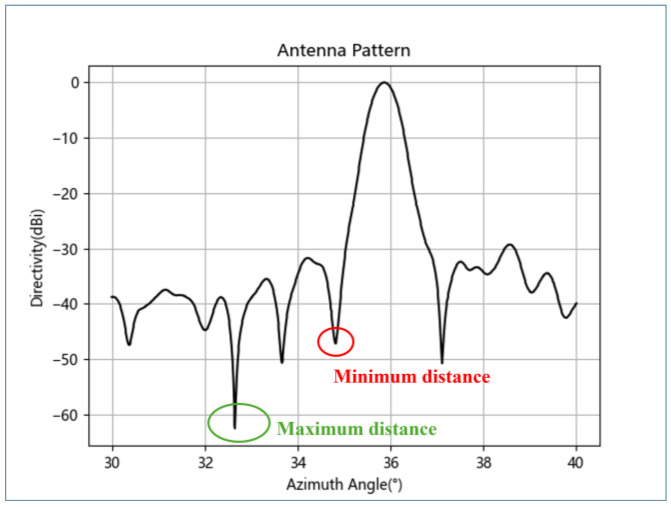
Radar antenna pattern.

When calibrating with a UAV-suspended metal sphere, to avoid interference from the UAV’s echo on the metal sphere and thus affect the calibration accuracy, the suspension rope should be long enough to ensure that the UAV is outside the main beam range of the radar. This can be achieved by examining the radar antenna pattern and determining the length of the suspension rope based on the maximum difference in amplitude between the main and side lobes (α); this is illustrated in Figure 4, where the length of the rope L_2_ is calculated according to Formula (16). Minimum distance represents the ropes corresponding to the nearest lobes, and Maximum distance represents the ropes corresponding to the farthest lobes.

(16) $$L_{2}=\alpha \pi R/180$$

To avoid the UAV and metal sphere in the same beam range, the length of the rope is usually taken as a distance of twice the beamwidth. For different radar bands, taking a 1.5° beamwidth as an example, so taking α = 3°, the typical values of rope length at different flying distances are shown in Table 2:

3. Determining Flight Route and Altitude.

Check the UAV settings, plan the flight route well. Since the UAV generates scattering signals of similar magnitude to the metal sphere, it should be prevented from entering the radar scanning beam during PPI and FIX scans. Additionally, the UAV’s flight altitude should be determined based on factors such as local airspace restrictions, antenna far-field range, rope length, and UAV performance.

#### 2.2.3. Synchronous Observation Experiment

1. Metal Sphere RCS Calculation.

**Figure 5:**
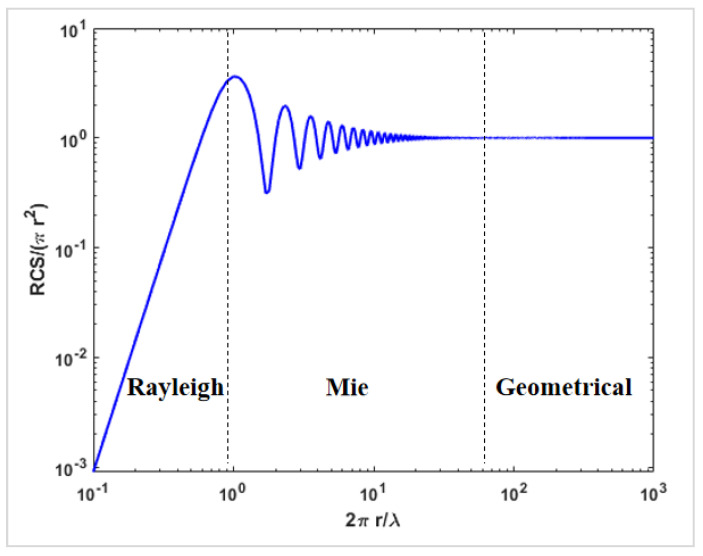
Normalized radar cross-section variation with wavelength.

Metal sphere calibration requires the precise calculation of the radar cross-section *σ*, commonly represented as RCS (radar cross-section), observed by the radar. The normalized scattering cross-section is primarily divided into the Rayleigh scattering region, Mie scattering region, and geometric optics scattering region [16]. The normalized scattering cross-section is a function of the dimensionless scattering parameter 2πr/λ (where r is the radius of the metal sphere, and λ is the radar wavelength) (Figure 5). When selecting the size of a metal sphere, it is important to control the relationship between diameter and wavelength. When the ratio is in the Rayleigh scattering region, it is difficult to control the stability of the RCS. However, when it is close to the geometric region, a stable RCS value can be obtained.

**Figure 6:**
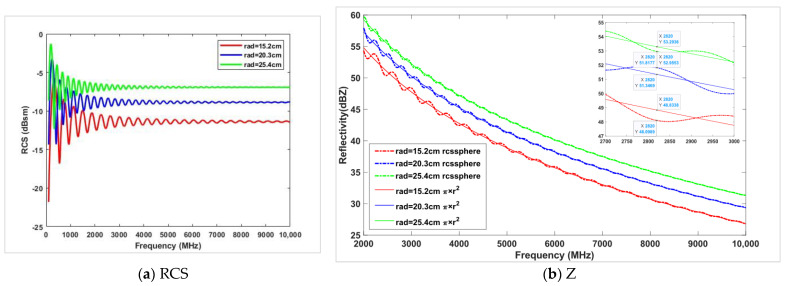
Trends of RCS and Z for metal spheres of different sizes as a function of frequency.

Typically, we use the simple formula π*r^2^ to represent the RCS. However, when the size of the metal sphere falls within the resonance region (Mie scattering region), its backscatter cross-section fluctuates. Figure 6a illustrates the simulated variation in the RCS with frequency for metal spheres of three sizes: 15.2 cm, 20.3 cm, and 25.4 cm in radius. Smaller metal spheres and lower radar frequencies result in larger fluctuations in the RCS. For obtaining high-precision measurement data, this study utilizes the normalization of the RCS using Bessel function integration [19,20], implemented with the rcssphere calculation function in MATLAB. Based on the parameters of the new-generation weather radar, the maximum reflectivity factor deviations caused by metal spheres of different sizes at various radar frequencies are computed using both the rcssphere function and the πr^2^ method.

(17) $$NRCS=\frac{\sigma }{\pi a^{2}}={\left(\frac{2}{fa}\right)}^{2}{\left|\sum_{n=1}^{\infty }\;{\left(-1\right)}^{n}\left(n+0.5\right)\left(b_{n}-a_{n}\right)\right|}^{2}$$

where

(18) $$a_{n}={\frac{j_{n}(fa)}{h_{n}^{\left(1\right)}(fa)}}^{2}$$

(19) $$b_{n}=\frac{faj_{n-1}(fa)-nj_{n}(fa)}{fah_{n-1}^{\left(1\right)}(fa)-nh_{n}^{\left(1\right)}(fa)}$$

in which a represents the radius of the metal sphere, λ represents the wavelength of the radar, f=2π/λ represents the wave number, $h_{n}^{\left(1\right)}(x)=j_{n}(x)+jy_{n}(x)$ represents the spherical Hankel function of the first kind, $j_{n}(x)$ represents the spherical Bessel function of the first kind, and $y_{n}(x)$ represents the spherical Bessel function of the second kind.

As shown in Figure 6b and Table 3, in the S-band region, 2700 MHz–3000 MHz is selected for inversion; the maximum error caused by a 15.2 cm radius metal sphere reaches 0.73 dB, while the error generated by larger metal spheres in the X-band range can be ignored (detailed statistical results are shown in Table 3). Therefore, based on accurate metal sphere RCS analytical calculation methods and considering the maximum payload capacity of the UAV, it is advisable to choose metal spheres with the largest possible radius to ensure the stability of the true value of the target during weather radar intensity calibration.

2. Calculating Relative Position.

**Figure 7:**
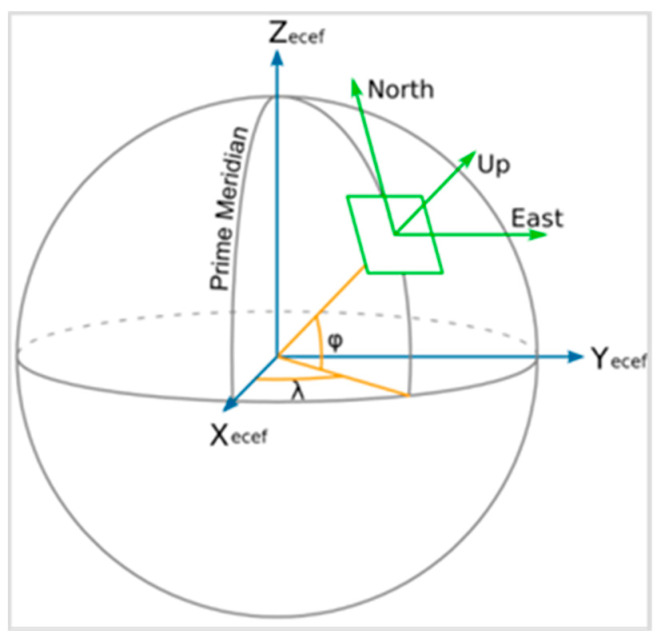
Diagram of Earth’s ellipsoidal coordinate system.

Using the real-time position of the metal sphere and the radar station information, the theoretical azimuth, elevation angle, and distance of the metal sphere relative to the radar can be calculated. Geodetic coordinates describe the spatial position of the target using latitude (*B*), longitude (*L*), and height (*H*), while the spatial rectangular coordinate system describes the spatial position of the target using *X*, *Y*, and *Z* [18]. As shown in Figure 7, the blue lines represent the geocentric rectangular coordinate system $P_{xyz}=\left(X,Y,Z\right);$ the orange lines represent the geodetic coordinate system $P_{BLH}=(B,L,H)$; and the green lines represent the ENU (East-North-Up) rectangular coordinate system $P_{ENU}=(x,y,z)$. Transforming P_BLH_ coordinates to P_ENU_ coordinates involves the following three steps:

Step 1: Convert BLH coordinates to XYZ coordinates. The conversion formulas are as follows [21]:

(20) $$\left.\left\{\begin{array}{l} X=(N+H)\cos\;B\cos\;L\\ Y=(N+H)\cos\;B\sin\;L\\ Z=(N(1-\varepsilon^{2})+H)\sin\;B \end{array}\right.\right.$$

where $N=\frac{a}{\sqrt{1-\varepsilon^{2}{sin}^{2}L}}$ represents the local curvature radius of the meridian circle, a denotes the semi-major axis of the Earth ellipsoid, and  ε signifies the Earth’s eccentricity.

Step 2: Convert geocentric rectangular coordinates XYZ to ENU (East-North-Up) rectangular coordinates xyz. The transformation is conducted using the matrix rotation formula as follows:

(21) $$\left[\begin{array}{c} x\\ y\\ z \end{array}\right]=\left[\begin{array}{ccc} -\sin\;\lambda_{r} & cos\;\lambda_{r} & 0\\ -sin\;\phi_{r}cos\;\lambda_{r} & -sin\;\phi_{r}sin\;\lambda_{r} & cos\;\phi_{r}\\ cos\;\phi_{r}cos\;\lambda_{r} & cos\;\phi_{r}sin\;\lambda_{r} & sin\;\phi_{r} \end{array}\right]\left[\begin{array}{c} X_{p}-X_{r}\\ Y_{p}-Y_{r}\\ Z_{p}-Z_{r} \end{array}\right]$$

where $\lambda_{r}$ is the longitude of the radar, $\phi_{r}$ is the latitude of the radar, $X_{r}$, $Y_{r}$, and $Z_{r}$ are the geocentric rectangular coordinates of the radar’s spatial position, and $X_{p}$, $Y_{p}$, and $Z_{p}$ are the geocentric rectangular coordinates of the target’s spatial position.

Step 3: Convert ENU (East-North-Up) rectangular coordinates to polar coordinates with respect to the station center, where R represents the distance, az represents the azimuth, and el represents the elevation angle.

(22) $$\left\{\begin{array}{l} R=\sqrt{x^{2}+y^{2}+z^{2}}\\ az=arctan\frac{x}{y}\\ el=arcsin\frac{z}{R} \end{array}\right.$$

3. Metal Sphere Target Acquisition.

**Figure 8:**
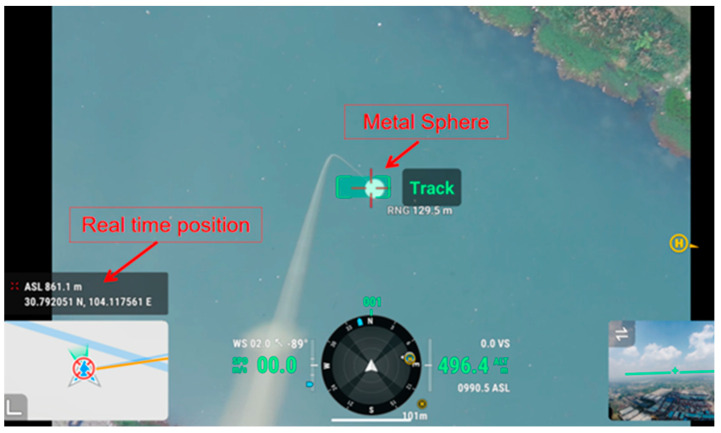
Laser rangefinder camera capturing metal sphere.

The efficient capture of the metal sphere first needs to obtain the real-time position of the metal sphere. Then, based on the three scanning modes of the radar RHI, PPI, and FIX, a high-success sphere search strategy of “coarse adjustment + fine adjustment + fixed gaze” is designed. Finally, command the UAV to suspend the metal sphere and fly along the radar radial direction (forward or backward), place the metal sphere in the distance center area, find the optimal target position, and obtain the best observation value. Figure 8 shows the operation interface of using high-precision RTK equipment and the Track function of a laser ranging camera to capture the real-time position of a metal sphere during field calibration above a lake.

#### 2.2.4. Accuracy Evaluation

Based on the high-precision RCS calculated value of the metal sphere, the relative distance, and the radar parameter values, the theoretical value of the radar’s observation of the metal sphere is calculated. From each set of observational data, select the data set with the smallest deviation from the theoretical value as the optimal value. Then, average the optimal values from multiple calibration results, with Z_average_, Z_DRaverage,_ and CC_average_ representing the final observation values.

The deviation calculation formulas for Z, Z_DR_, and CC are as follows:

(23) $$∆Z=Z_{Observed\;Value}-Z_{theoretical\;value}$$

(24) $$∆Z_{DR}={Z_{DR}}_{Observed\;Value}-{Z_{DR}}_{theoretical\;value}$$

(25) $$∆CC={CC}_{Observed\;Value}-{CC}_{theoretical\;value}$$

Refer to the requirements of the “S-band New Generation Weather Radar Functional Specification Requirements” and “Weather Radar Calibration Methods and Regulations (Trial)” issued by the China Meteorological Administration (CMA) for reference. The calibration accuracy indicators for the metal sphere are shown in Table 4:

Using data from metal spheres allows for the fitting of radar antenna and transmitter parameters. By selecting data from the distance range where the metal sphere is located, a parabolic curve fitting using the least squares method can simulate the numerical value of the radar beamwidth, with higher accuracy compared to traditional methods based on the maximum amplitude values. Since the metal sphere’s echo undergoes bidirectional amplitude modulation by the antenna, a round-trip echo implies the need to calculate a 6 dB beamwidth to represent a “single trip 3 dB beamwidth”. Similarly, using data from adjacent distance ranges before and after the metal sphere, the radar pulse width can also be fitted. If the fitting results match the actual radar parameters, it indicates the effectiveness of the radar in observing the scattering characteristics of the metal sphere. To validate the calibration results, this study uses the same weather processes observed by the reference radar at Changsha Meteorological Radar Calibration Center before and after calibration with the metal sphere, cross-validating the radar’s actual observation products before and after metal sphere calibration, quantitatively evaluating the reliability and accuracy of the metal sphere calibration from the perspective of basic data.

## 3. Experimental Area and Data Source

### 3.1. Introduction to the Experimental Area

**Figure 9:**
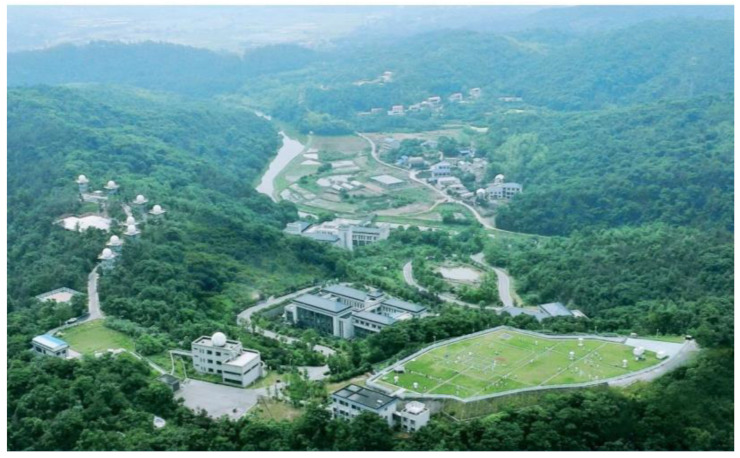
Changsha Meteorological Radar Calibration Center.

**Figure 10:**
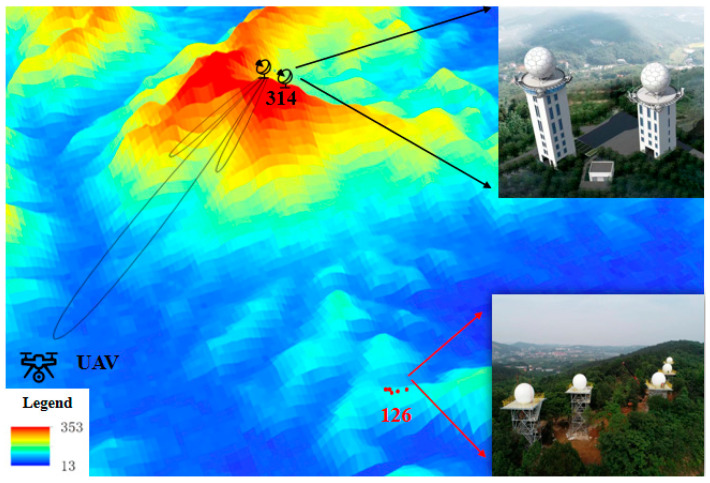
Radar calibration sites 314 and 126.

Changsha Meteorological Radar Calibration Center is the only radar calibration base in China (Figure 9). It relies on the terrain advantage of Mount Lianhua and has established calibration equipment such as an S-band high-precision dual-polarization reference radar, far-field calibration system, and comprehensive calibration instrument, capable of testing 54 key parameters of weather radars. Multi-type S, C, and X-band radars are located at radar calibration sites 314 and 126 (314 and 126 named according to their respective altitudes). This study focuses on the calibration of the C-band dual-polarization radar (BC1) and cross-validates the radar calibration accuracy with the co-located S-band reference radar (SSR). The SSR radar undergoes a regular high-precision calibration of 54 technical indicators, combining dynamic and static methods to ensure the accuracy of its observation data. The BC1 radar is located 40 m away from the SSR radar, with a tower height 14 m lower than that of the SSR radar; thus, the region where the SSR radar tower obstructs the BC1 radar (142°–166°) has been mitigated. Combined with the Digital Elevation Model (DEM) shown in Figure 10, all radars are situated at high altitudes in mountainous areas, with relatively minimal influence from terrain obstruction.

### 3.2. Radar Parameters

First, the SSR reference radar and the BC1 radar to be calibrated underwent routine calibration for various static parameters. For this metal sphere field test, three scanning modes were employed for all radars. Considering the beam coverage and to save flight time, the RHI and PPI scanning ranges were set to ±8°. Appropriate scanning speeds, pulse repetition frequencies, and other parameters were selected. All filters were turned off to collect data before filtering. The specific scanning parameters are as follows (Table 5):

### 3.3. UAV, Laser Rangefinder, and Metal Sphere Parameters

This experiment used a professional DJI Matrice 300 RTK UAV with centimeter-level positioning accuracy. A DJI H20 camera with high-precision laser ranging capabilities was installed on the UAV, and a standard metal sphere of appropriate size was suspended below the UAV. The specific parameters are shown in Table 6 below:

## 4. Results

### 4.1. An Analysis of the Metal Sphere Position

**Figure 11:**
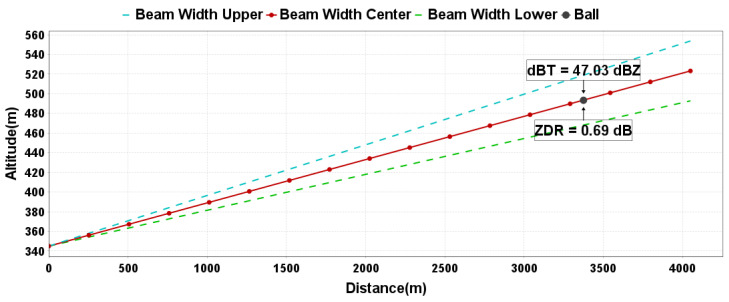
Inversion of metal sphere position.

In this study, an outdoor experiment for S and C band radars was conducted using the largest available metal sphere (with a radius of 25.4 cm) for calibration. Figure 11 shows the process of metal sphere calibration. By utilizing RTK equipment and a laser rangefinder camera to obtain the precise real-time position of the metal sphere, its location can be mapped onto the radar beam height and range bin. It is visually evident that the metal sphere is significantly shifted towards the previous range bin. The observed Z value is 47.03 dB, while the theoretical Z value is 50.71 dBZ, resulting in a deviation of 3.7 dB. Additionally, the observed Z_DR_ value is 0.69 dB, which also shows a considerable deviation.

**Figure 12:**
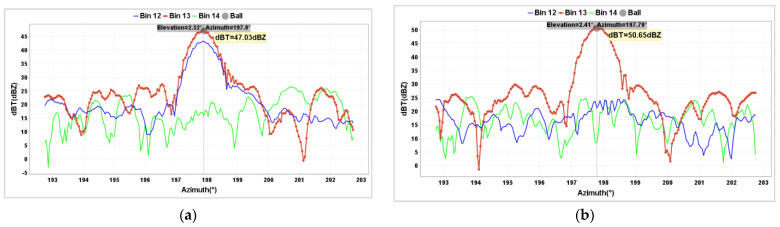
Metal sphere not at (**a**) and at (**b**) the radar range gate center position.

By analyzing the data of adjacent range gates for the metal sphere, it was found that when the metal sphere is located at the center of the range gate, the echo values of the adjacent front and rear range gates should be relatively close and stable and significantly smaller than the echo intensity value of the range gate where the metal sphere is located. Conversely, the data of the adjacent front and rear range gates show greater fluctuations. Figure 12a analyzes the data from Figure 11. It can be seen that due to the metal sphere being close to the 12th range gate, it causes the 12th range gate to produce an echo intensity value equal to that of the 13th range gate. By having the UAV move the metal sphere approximately 50 m away from the radar, placing the metal sphere at the center of the 13th range gate, the elevation angle decreased by 0.1°, and the echo intensity value of the 12th range gate significantly decreased, roughly matching the data of the 14th range gate. At this point, the observed Z value was 50.65 dBZ (Figure 12b), with a deviation of only −0.06 dB. Additionally, Z_DR_ and CC values should also be observed when the metal sphere is at the center of the range gate.

### 4.2. First Metal Sphere Calibration

**Figure 13:**
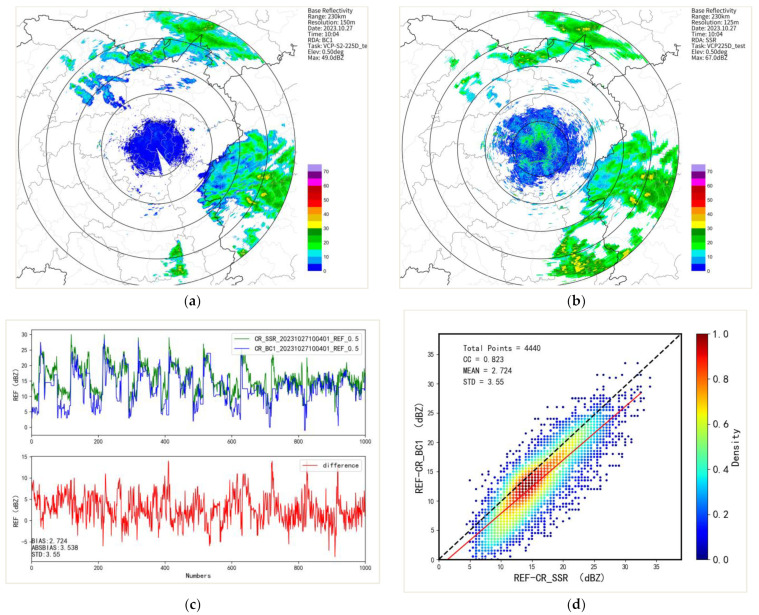
A comparison of original data from two radars at the same location. (**a**) shows the original echo of the BC1 radar; (**b**) shows the original echo of the SSR radar; (**c**) shows a line graph of the consistency of two radar echoes; (**d**) shows a scatter plot of the consistency of two radar echoes.

On 27 October 2023, during the observation of a precipitation event, two radar systems underwent a comparative analysis using the co-located radar comparison algorithm to assess the consistency of echo signals between the tested radar and the reference radar. It was found that the echo positions and areas of both radars were generally consistent, with a correlation coefficient exceeding 0.82 and a standard deviation of 3.55. However, the reflectivity factor of the BC1 radar was on average 2.72 dB weaker than that of the reference SSR radar (Figure 13).

**Figure 14:**
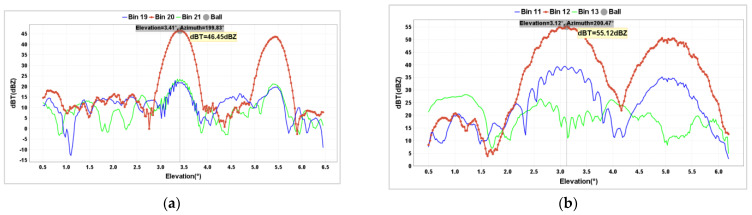
Calibration results of Z values for BC1 (**a**) and SSR (**b**) radars on 6 November 2023.

Therefore, on 6 November 2023, the first calibration of the BC1 radar with the metal sphere was conducted (Figure 14a), and the SSR reference radar also underwent simultaneous observation with the same sphere (Figure 14b). It was found that the observed value of the BC1 radar was 3.06 dB lower than the theoretical value, while the observed value of the SSR radar with the same sphere deviated from the theoretical value by only 0.03 dB (Table 7).

This shows that the BC1 radar underestimated the precipitation echo and the standard metal sphere by about 3 dB each. To explore the reason for the low echo, the antenna beamwidth and gain of the BC1 radar were measured using the antenna far-field test system at the Changsha Calibration Center, revealing that the use of radar factory antenna test data caused a 2.31 dB underestimation of the echo. In addition, the transmitter power test did not set the loss value according to the actual situation, resulting in a 0.59 dB underestimation of the echo. The combination of the two led to a total weakening of the BC1 radar echo intensity by 2.9 dB. The BC1 radar parameters were calibrated based on the on-site measurement data.

### 4.3. Post-Calibration Data Authenticity Verification

#### 4.3.1. The Second Metal Sphere Calibration

On 1 December 2023, the BC1 radar underwent its second metal sphere calibration. For this calibration, retested and recalibrated radar parameter data were used. The position of the metal sphere was similar to that during the first calibration. The parameters of the radar, metal sphere, and UAV are listed in Table 8.

**Figure 15:**
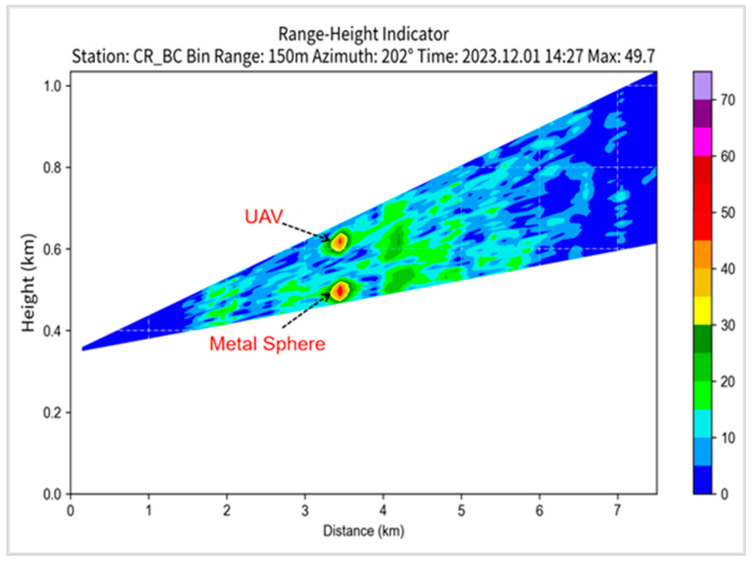
RHI scan result of metal sphere calibration of BC1 radar on 1 December 2023.

**Figure 16:**
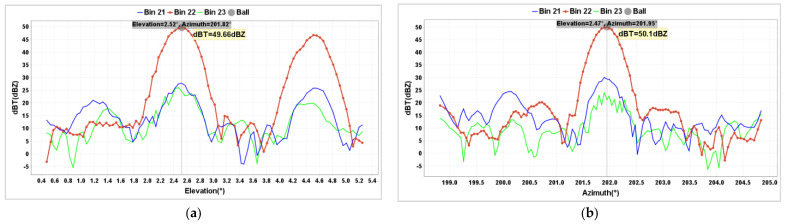
Z value RHI (**a**) and PPI (**b**) scan calibration results of BC1 radar on 1 December 2023.

The unmanned aircraft took off at an altitude of 119 m above sea level, ascended 483 m, and then reached an altitude of 602 m. To ensure no interference between the unmanned aircraft and the metal sphere’s echoes, a 112 m long rope was used in the experiment, placing the metal sphere at an altitude of 490 m. Initially, an RHI scan was conducted. Near BC1 radar azimuth 202°, at a distance of 3.3 km, and an altitude of 490 m, a circular echo with a strong center and weakening edges was observed, indicating the radar echo generated by the metal sphere. In the area near an altitude of 600 m directly above, a strong echo region was observed, indicating the radar echo generated by the unmanned aircraft. These two echoes were completely separate and did not interfere with each other (Figure 15). Data analysis revealed that the metal sphere was observed at the 22nd distance bin of the radar. At this point, the curve showed a double-peak shape, with the high-elevation peak representing the unmanned aircraft and the low-elevation peak representing the metal sphere (Figure 16a). During PPI scanning, the curve appeared as a single-peak state, indicating that the echo from the unmanned aircraft did not enter the main radar beam range (Figure 16b).

**Figure 17:**
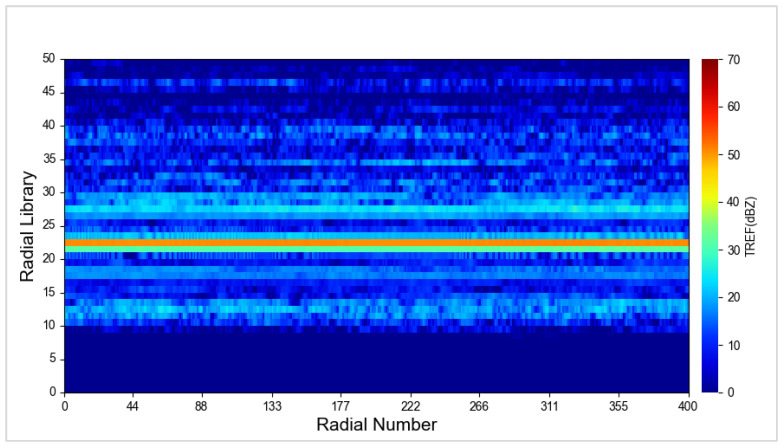
Z value calibration results of BC1 radar FIX scan on 1 December 2023.

After completing RHI and PPI scans, the radar obtains precise pitch and azimuth angles for the metal sphere. The radar is then directed to the metal sphere to execute FIX directional scanning. Continuous echo data from the metal sphere are obtained. If the metal sphere is positioned between the beam and the distance bins with minimal oscillation, the echo from the metal sphere appears as a smooth straight line. The echo intensity from adjacent distance bins also remains relatively stable, with values significantly lower than that of the metal sphere. The orange line in Figure 17 represents the continuous echoes observed by the radar during the FIX scan.

**Figure 18:**
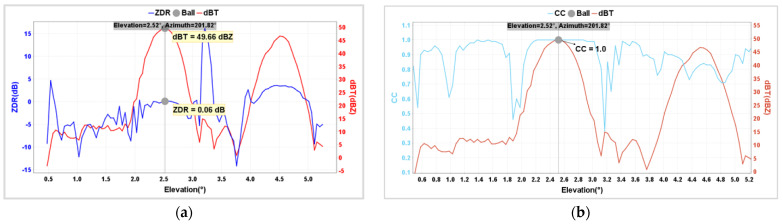
Calibration results of Z_DR_ (**a**) and CC (**b**) for BC1 radar on 1 December 2023.

Figure 18 shows that during RHI and PPI scans, in the regions where the Z value rises, falls, and fluctuates, there is significant jitter in both Z_DR_ and CC values due to incomplete alignment with the metal sphere and insufficient backscatter filling. The strongest backscatter from the metal sphere occurs at the point where the strongest echo is produced, and the Z_DR_ and CC observed values obtained at this point are more representative.

Table 9 presents the calibration results of metal spheres under different scanning modes on 1 December 2023. The theoretical reflectivity factor of the metal sphere is 50.06 dBZ. The observed reflectivity values by the radar under RHI, PPI, and FIX scanning modes are 49.66 dBZ, 50.10 dBZ, and 50.65 dBZ, respectively, with corresponding differences of −0.40 dBZ, 0.04 dBZ, and 0.59 dBZ. The average deviations are 0.08 dBZ. Similarly, the average deviations for Z_DR_ and CC are −0.06 dB and 0, respectively. It can be concluded that the calibration results of the metal sphere this time are satisfactory, fully meeting the requirements of weather radar technical specifications.

#### 4.3.2. Cross-Validation of Radar at Same Site

**Figure 19:**
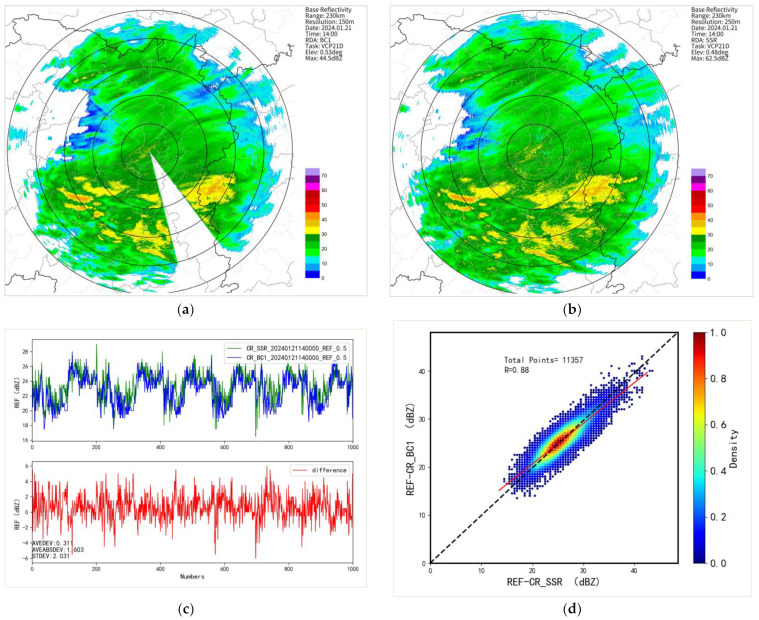
A co-location comparison analysis of two radars at the same site at 14:00 (UTC) on 21 January 2024. (**a**) shows the original echo of the BC1 radar; (**b**) shows the original echo of the SSR radar; (**c**) shows a line graph of the consistency of two radar echoes; (**d**) shows a scatter plot of the consistency of two radar echoes.

Utilizing a widespread snowfall event on 21 January 2024, the accuracy of BC1 radar data was validated. Figure 19 analyzes the volumetric scan data observed by the BC1 and SSR radars at the same site at 14:00 (UTC). The average deviation was 0.31 dB, the standard deviation was 2.03 dB, and the correlation coefficient was 0.88. Table 10 presents the comparison results of continuous 1 h (10 volumetric scans) co-located observation data from both radars during this snowfall event. The average deviation was 0.32 dB, the standard deviation was 2.17 dB, and the correlation coefficient was 0.85. It can be observed that the echo structure and intensity between the two radars were generally consistent.

#### 4.3.3. Radar Key Parameter Validation

**Figure 20:**
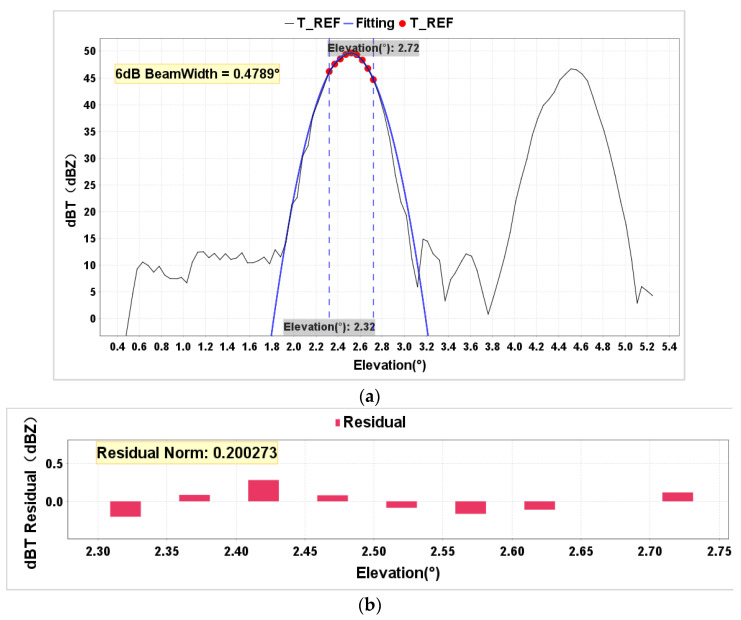
A simulation of beamwidth. (**a**) shows a 6 dB beamwidth simulation of metal sphere data scanned by RHI; the red dots represent the measured data, and the blue lines represent the fitted curves; (**b**) a residual analysis of the data participating in the fitting (elevation 2.32°–elevation 2.72°).

**Figure 21:**
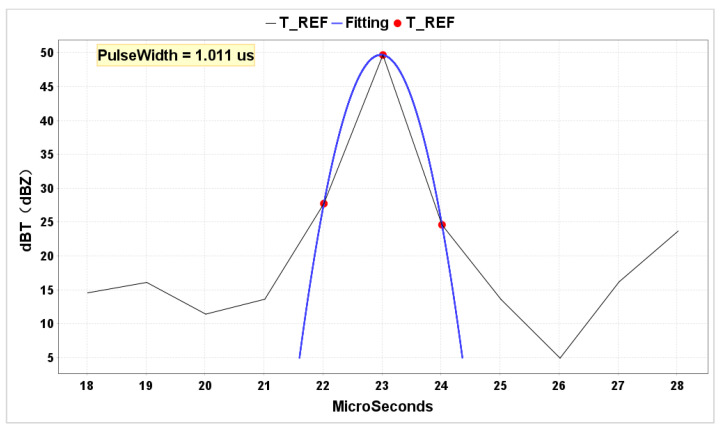
A simulation of pulse width. The red dots represent the measured data, and the blue lines represent the fitted curves.

The radar’s key parameters were tested using the echo data of metal spheres from the RHI scan on 1 December 2023. The fitted value of the 6 dB beamwidth was 0.4789° (Figure 20), which is in close agreement with the 0.48° beamwidth value measured during antenna far-field system testing. Additionally, the average residual was 0.2 dB, indicating a small average difference between the model’s predicted values and the observed values. Converting radar radial data to the time dimension, the radar pulse width was fitted using the data from the radial where the metal sphere was located, as well as the adjacent radial data before and after (Figure 21). The fitted value obtained was 1.011μs, which is consistent with the instrument’s measured value of 1μs. Since only three points were used for the parabolic fitting, the residual was 0.

## 5. Conclusions

This study established a detailed method for weather radar intensity calibration and field-testing procedures using a UAV-suspended metal sphere, summarizing various aspects such as the scattering characteristics of the metal sphere, theoretical values of field calibration, target capture and observation, optimal positioning analysis, and accuracy evaluation methods. The results of two metal sphere calibration experiments demonstrate that metal sphere calibration, as an absolute calibration method for the entire chain, can be used to check the accuracy of radar antenna pointing and observation products with high frequency and periodicity. Moreover, the calibration accuracy of Z and Z_DR_ can reach 0.5 dB and 0.2 dB, respectively. The combined application of high-precision RTK UAVs and laser ranging cameras effectively improves the capture speed and alignment accuracy of the metal sphere. By analyzing the characteristics of adjacent distance bin echoes of the metal sphere, it was found that when the metal sphere is placed at the center of the radar radial distance bin, the radar can obtain the most accurate calibration data, and the results of parameters such as radar beamwidth and pulse width are relatively reliable.

The metal sphere calibration method proposed in this paper will provide technical accumulation for the metal sphere calibration method and experimental design of phased array radar. Considering that the uncertainty of the metal sphere’s attitude in the air increases the difficulty of beam alignment, future exploration will focus on establishing a method for multiple UAVs to collaboratively suspend metal spheres to reduce errors caused by the pendulum effect and rotation of the sphere. Furthermore, as the metal sphere serves as a point target that can fit the radar antenna pattern, the next step will be to explore the establishment of multi-layer PPI scanning modes to obtain the peak value of the antenna pattern and 3D inversion results of the metal sphere.

## Figures and Tables

**Table 1:** Far-field distance corresponding to different radar bands.

Radar Band	λ/cm	D/m	L/m
S-Band (2.85 GHz)	10.5	8.4	1344
C-Band (5.5 GHz)	5.45	4.5	743
X-Band (9.45 GHz)	3.2	2.4	360

**Table 2:** Typical rope length values for different radar bands.

Band	L/m	R/m	L_2_/m
S	1344	3000	157
C	743	2000	105
X	360	1000	53

**Table 3:** Statistics of maximum reflectivity factor errors.

Radius/cm	Difference/dB		
	S-Band	C-Band	X-Band
15.2	0.73	0.26	0.10
20.3	0.47	0.16	0.05
25.4	0.34	0.10	0.03

**Table 4:** Calibration accuracy indicators for metal sphere.

Product	Radar Technical Specifications	Accuracy Specifications for Metal Sphere Calibration
Z	±1 dB	±0.5 dB
Z_DR_	±0.2 dB	±0.2 dB
CC	≤0.01	≤0.01

**Table 5:** Radar parameters.

Parameter Name	Reference Radar SSR	Calibrated Radar BC1
Scan Mode	RHI, PPI, FIX	
Pulse Accumulation	64	64
Operating Wavelength (cm)	10.54	5.31
Pulse Repetition Frequency (Hz)	1024	1024
Pulse Width (μs)	1.57	1.0
Horizontal Beamwidth (°)	0.91	0.48
Vertical Beamwidth (°)	0.86	0.43
Pulse Duration (m)	250	150

**Table 6:** Main technical parameters of calibration equipment.

Calibration Equipment	Photo	Performance Indicators	Parameters
Matrice 300 RTK UAV (Made by DJI, Shenzhen, China)	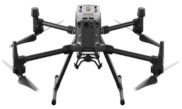	Operating frequency	2.4000–2.4835 GHz
		Maximum endurance	55 min
		Maximum horizontal flight speed	17 m/s
		Maximum altitude	5000 m
		Maximum wind resistance	15 m/s (Wind force scale 7)
		RTK positioning accuracy	The maximum tolerable wind speed during takeoff and landing is 12 m/s.
		GNSS	1 cm + 1 ppm (Horizontal)
H20 Laser Rangefinder Camera (Made by DJI, Shenzhen, China)	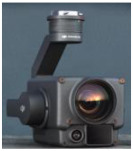	Wavelength	905 nm
		Accuracy of laser rangefinder	±(0.2 m + D × 0.15%), where D represents the distance to the vertical reflecting surface
		Measurement range	3–1200 m
Metal Sphere (Made by CENTURY METAL SPINNING, Illinois, USA)	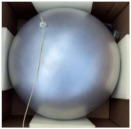	Radius	25.4 cm
		Weight	2.5 kg
		Roundness	99.88%

**Table 7:** The parameters of both radars and the metal sphere during the first calibration.

Parameters	Radar BC1	Radar SSR
λ (cm)	5.31	10.54
θ (°)	0.54	0.91
Φ (°)	0.51	0.86
τ (μs)	1	1.57
R (m)	2925.10	2904.35
r (cm)	25.40	25.40
Theoretical value (dBz)	49.67	55.15
Observed value (dBz)	46.45	55.12
Difference (dB)	−3.22	−0.03

**Table 8:** The parameters of the BC1 radar and metal sphere during the second calibration.

Equipment	Parameters	Value
Radar BC1	λ (cm)	5.31
	θ (°)	0.48
	Φ (°)	0.43
	τ (μs)	1
	R (m)	3231.31
Metal Sphere	r (cm)	25.40
	L_2_ (m)	112
UAV	h_3_ (m)	119
	h_2_ (m)	483

**Table 9:** The results of the second metal sphere calibration.

Product	Z (dBZ)			Z_DR_ (dB)			CC		
Observed Value	RHI	PPI	FIX	RHI	PPI	FIX	RHI	PPI	FIX
	49.66	50.10	50.65	0.06	−0.13	−0.11	1	1	1
Theoretical Value	50.06			0			1		
Difference	−0.40	0.04	0.59	0.06	−0.13	−0.11	0	0	0
Average Deviation	0.08			−0.06			0		

**Table 10:** Statistical comparison of co-located data from two radars for one hour on January 21, 2024.

Comparison Count	Time of Comparison	Sample Size (Items)	Mean Deviation (dB)	Standard Deviation (dB)	Correlation Coefficient
1	20240121133000	15,352	0.62	2.11	0.88
2	20240121133601	25,482	0.55	2.04	0.86
3	20240121134201	18,862	0.35	2.04	0.86
4	20240121134801	17,559	0.31	1.96	0.87
5	20240121135401	22,524	0.39	1.97	0.86
6	20240121140000	11,357	0.31	2.03	0.88
7	20240121140600	3306	0.18	2.57	0.75
8	20240121141200	5742	0.02	2.3	0.72
9	20240121141800	14,729	0.16	2.41	0.87
10	20240121142400	19,003	0.33	2.26	0.9
Mean Deviation		15,391.6	0.32	2.17	0.85

## Data Availability

All code, data, and materials included in this research are available upon request from the corresponding author.
